# How the World Council of Optometry produced new guidelines for myopia management

**Published:** 2023-01-30

**Authors:** Peter Hendicott, Sandra S Block

**Affiliations:** Adjunt Associate Professor: School of Optometry and Vision Science, Queeensland University of Technology, Australia and President: World Council of Optometry, USA.; Professor Emeritus, Illinois College of Optometry and President-Elect: World Council of Optometry, USA.

## Abstract

The World Council of Optometry (WCO) relies on scientific practices and high quality research to develop eye care guidelines.

The World Council of Optometry (WCO) is an international organisation focused on promoting eye health and vision care as a human right; as part of its mission, the WCO also seeks to advance the role of optometry in health care through advocacy, education, and collaboration.

At WCO, we feel strongly that any information we share with eye care providers worldwide, or in our advocacy, needs to be based on published, scientific evidence that has been vetted (peer reviewed) by experts in the field. However, scientific publications are often inaccessible to practicing clinicians for many reasons.

One of our goals is therefore to help translate the outcomes of well-designed studies – published in reputable, peer-reviewed scientific journals – into guidelines for clinical care that can used by optometrists.

## Myopia guidelines: an example

Myopia is a rapidly growing public health issue in many countries. In 2021, WCO passed a resolution that highlighted myopia and the need to both rethink how eye care providers identify children at risk of developing myopia, and how they can better manage myopia in children. The resolution defined an evidence-based standard of care for myopia with three main components:

1. **Mitigation.** Optometrists educating and counselling parents and children, during early and regular eye examinations, on lifestyle, dietary, and other factors that prevent or delay the onset of myopia.
2. **Measurement.** Optometrists evaluating the myopic status of a patient during regular, comprehensive vision and eye health examinations, i.e., measuring refractive error and axial length whenever possible.
3. **Management.** Optometrists addressing patients’ current needs by correcting myopia, while also providing evidence-based interventions (e.g., contact lenses, spectacles, and/or pharmaceuticals) that slow the progression of myopia and offer improved quality of life and better eye health, both now and in the future.

The resolution also advised optometrists to incorporate the above standard of care for myopia management within their practice, and therefore to not only correct vision, but also to include in their work, public education and early and frequent discussions with parents that explain:

- what myopia is
- lifestyle factors that may impact myopia
- the available approaches that can be used to manage myopia and slow its progression.

In order to support optometrists to implement the new standard of care, WCO, in collaboration with CooperVision, invited a group of experienced clinicians and educators to help identify and evaluate high quality current evidence on myopia and develop *Standard of Care Guidelines for Myopia Management*,^1^ a set of practical guidelines and tools available online. Each of the practical recommendations (for mitigation, measurement, and management) is accompanied by a list of the research publications that informed its development.

The group used the following criteria when deciding whether to include a research study in their deliberations:

- The study was approved by an institutional ethics committee or review board (see panel).
- The research question is clearly defined, so that the study provides a quantitative outcome for a specific question.
- The study design and methodology is set out clearly, so that the investigators could strictly follow the guidelines, eliminating as many confounding factors as possible and enabling others to replicate their methods.
- The data is collected and recorded in a clear, consistent manner, so that the analysis of the data yields clear outcome measures that answer the original research question.
- The results and conclusions are based on the evidence.
- The characteristics of subjects included in the studies are fully described, so that there is a clear understanding about the relevance of the outcomes to particular groups.
- Authors or principal investigators state the limitations of their findings so that the results can be interpreted correctly.

As the WCO does not directly conduct investigative research, we rely on the work of dedicated researchers who are conducting impactful studies that inform how we can best serve our patients. We will continue to look to them for ways to reduce the impact of visual impairment and blindness globally.

**Figure:**
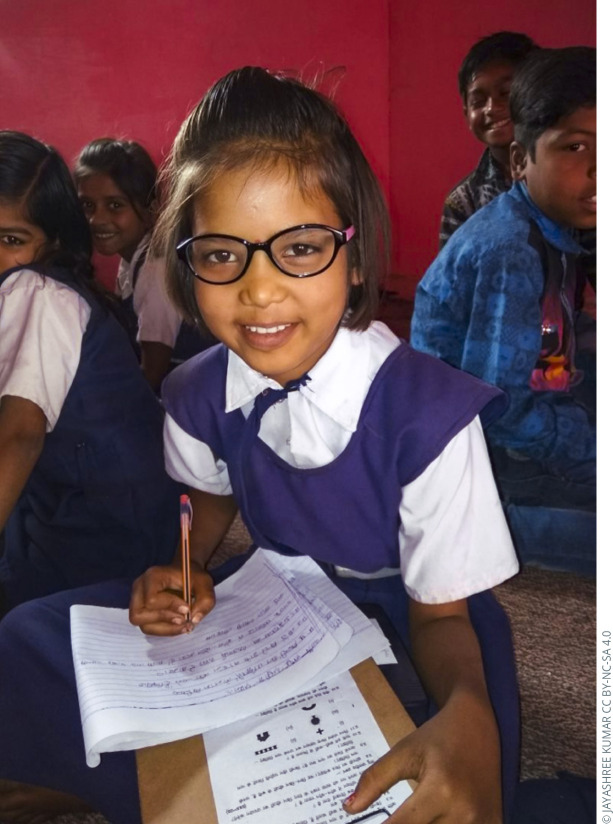
Myopia is a rapidly growing public health issue in many countries. india

**Ethics**

When attempting to answer clinical practice questions, as in this example of myopia, WCO strives to adhere to the principles of the Declaration of Helsinki – protection of the health, wellbeing, and rights of patients, including those involved in medical research.^2^ We therefore require that human subjects are protected during any research that we support or report on. This includes ensuring that any personal, medical, educational, or other private information is not shared without the written authorisation of the individual.

WCO therefore requires that any research studies we report have been approved by a reputable institutional review board or ethics committee. These boards or committees are typically made up of experts and community members who review the design of the study to ensure that it is appropriate for the hypothesis posed by the principal investigator and that appropriate consideration has been given to the principles of the Declaration of Helsinki.
